# Characterising the Role of GABA and Its Metabolism in the Wheat Pathogen *Stagonospora nodorum*

**DOI:** 10.1371/journal.pone.0078368

**Published:** 2013-11-12

**Authors:** Oliver Mead, Eli Thynne, Britta Winterberg, Peter S. Solomon

**Affiliations:** Plant Sciences Division, Research School of Biology, The Australian National University, Canberra, Australia; Rutgers University, United States of America

## Abstract

A reverse genetics approach was used to investigate the role of γ-aminobutyric acid metabolism in the wheat pathogenic fungus *Stagonospora nodorum*. The creation of mutants lacking *Sdh1*, the gene encoding succinic semialdehyde dehydrogenase, resulted in strains that grew poorly on γ-aminobutyric acid as a nitrogen source. The *sdh1* mutants were more susceptible to reactive oxygen stress but were less affected by increased growth temperatures. Pathogenicity assays revealed that the metabolism of γ-aminobutyric acid is required for complete pathogenicity. Growth assays of the wild-type and mutant strains showed that the inclusion of γ-aminobutyric acid as a supplement in minimal media (i.e., not as a nitrogen or carbon source) resulted in restricted growth but increased sporulation. The addition of glutamate, the precursor to GABA, had no effect on either growth or sporulation. The γ-aminobutyric acid effect on sporulation was found to be dose dependent and not restricted to *Stagonospora nodorum* with a similar effect observed in the dothideomycete *Botryosphaeria* sp. The positive effect on sporulation was assayed using isomers of γ-aminobutyric acid and other metabolites known to influence asexual development in *Stagonospora nodorum* but no effect was observed. These data demonstrate that γ-aminobutyric acid plays an important role in *Stagonospora nodorum* in responding to environmental stresses while also having a positive effect on asexual development.

## Introduction

*Stagonospora nodorum* is the causal agent of stagonospora nodorum blotch, a significant disease of wheat in many parts of the world [1]. It is has now been established that the wheat pathogen *S. nodorum* causes disease through the secretion of small secreted proteins (effectors) that interact with dominant susceptibility host genes leading to cell death and disease [2]. These studies have fundamentally advanced our understanding of the interaction and provided significant insight into how this necrotrophic pathogen causes disease [3].

Whilst this effector model has now been established as the means by which the pathogen initiates disease, there still remains much to learn about how the fungus grows, develops and reproduces in the host. Several studies to date have identified key pathogen metabolic pathways required to complete the infection cycle. For example, reverse genetics approaches have demonstrated that the metabolism of mannitol, particularly through the activity of mannitol 1-phosphate dehydrogenase (Mpd1) is essential for asexual sporulation [4], [5], [6]. Mutants lacking the *Mpd1* gene were unable to sporulate, either *in vitro* or *in planta*, unless supplied with exogenous mannitol. A similar approach identified that trehalose also plays a key role in sporulation [7]. Disruption of a trehalose 6-phosphate synthase resulted in decreased sporulation that could be restored in the presence of added trehalose. The role of either of these primary metabolites on sporulation remains elusive although it was clearly demonstrated that the metabolism of these compounds, rather than simply their presence, was required for sporulation.

In a more recent study, a short-chain dehydrogenase, Sch1, was identified as being negatively regulated in *S. nodorum* strain lacking the Gα subunit Gna1 [8]. Subsequent disruption of the *Sch1* gene resulted in a strain that was unable to produce pycnidiospores, although abnormal pycnidia were differentiated. Metabolite analysis of the mutant strain showed that neither mannitol nor trehalose levels were affected. Indeed primary metabolism appeared relatively unaffected by the *Sch1* mutation. Metabolite profiling though did reveal that the secondary metabolite, alternariol, was present at very high levels in the mutant leading to speculation as to its role in asexual differentiation. Subsequent studies on *S. nodorum* sporulation-deficient strains have consistently identified changes in abundance in alternariol suggesting that it, and/or its associated pathway, play an integral role in sporulation [9].

**Figure 1:**
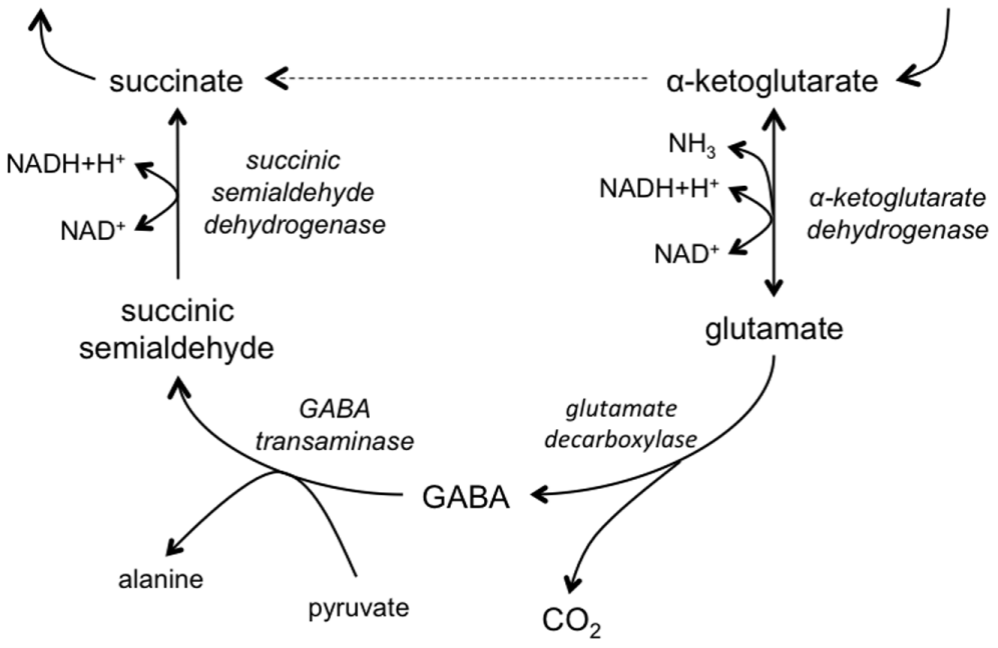
The GABA shunt.

These studies highlight the critical role of specific primary metabolic processes in the development and differentiation of *S. nodorum*. However there are many other pathways whose roles aren't clear during either development or pathogenicity. One such pathway is the γ-aminobutyric acid (GABA) shunt (Figure 1). This pathway is a bypass of the TCA cycle from α-ketoglutaric acid to succinic acid via glutamate, GABA and succinic semialdehyde. In contrast to the TCA cycle (between α-ketoglutaric acid and succinic acid), the GABA shunt results in no net gain in NADH or ATP, leaving many to question its biological role.

GABA itself is a well described negative neurotransmitter in the central and peripheral nervous system of vertebrates and some non-vertebrates [10]. However the role of GABA and its metabolism in other systems is less well understood. In plants, GABA has been shown to accumulate under different forms of stress [11], [12], [13], [14]. Mutational studies in *Arabidopsis thaliana* have demonstrated that the GABA pathway plays a role in responding to osmotic stress, as well as high temperature and light stress [15]. GABA has also been found to accumulate in tomato leaves during a compatible interaction with *Cladosporium fulvum*, leading to speculation that the pathogen was manipulating the host to produce a preferred nitrogen source [16]. However there has been no further evidence to support this.

The role of GABA and/or its metabolism in fungi is even less clear although it is well known that GABA is a suitable nitrogen source for many fungi [17]. A recent study in *Magnaporthe oryzae* identified a gene encoding succinic semialdehyde dehydrogenase (MoSSADH) as being important for pathogenicity [18]. Characterisation of the MoSSADH mutants found that the gene was required for appressorium-like penetration, invasive growth and normal development and conidiation. The mutants were also highly sensitive to H_2_O_2_ and displayed attenuated peroxidase and laccase activities. The bases of these phenotypes and the role of MoSSADH though were not elucidated.

In this study, we have focused on understanding the role of GABA catabolism on the pathogenicity and development of *S. nodorum* through the inactivation of the succinic semialdehyde dehydrogenase gene, *Sdh1*. The gene encoding succinic semialdehyde dehydrogenase was selected to allow a direct comparison of its role in a necrotrophic pathogen compared to the hemibiotrophic *M. oryzae*. This study has revealed various important roles for succinic semialdehyde dehydrogenase in *S. nodorum*, and like previous studies have done [7], has highlighted the significant differences that exist in the metabolic requirements of these different pathogens during infection. These data have also shed light on the novel role of GABA promoting asexual sporulation in *S. nodorum*.

## Materials and Methods

### Fungal strains and growth conditions

*Stagonospora nodorum* was maintained and grown as previously described [19]. Minimal media consists of 30 mM sucrose, 2 g/L NaNO_3_, 1 g/L NaH_2_PO_4_ and 10 mL trace stock solution, pH 6. For the supplementation assays, the chemicals (e.g. GABA) were added to media prior to pH adjustment. All strains were routinely grown under at 12 hr light/dark cycle at 22°C unless otherwise stated. Oxidative stress growth assays were undertaken by the addition of analytical grade H_2_O_2_ to agar plates to the final concentrations stated in the Results.

The dothideomycete *Botryosphaeria* sp. was kindly provided by Dr. Hugh Wallwork from the South Australian Research and Development Institute. The strain was grown on Botryosphaeria growth medium (BGM), which was prepared by adding 15 g of oats to 200 mL of deionised water and bringing to the boil in a microwave. To this, 40 g of fresh wheat leaves ground with a mortar and pestle in a small amount of water were added. The resulting liquid was then filtered and brought to 1 L with deionised water. 1.5% agar was added to the media prior to autoclaving. When used, 1 mM GABA was added prior to autoclaving.

### Sporulation assays

For *S. nodorum* sporulation assays, agar plates were inoculated in the centre with 10 µL of 10^6^ spores/mL, allowed to dry and then wrapped in micropore tape (3M). The plates were incubated for 20 days prior to being flooded with 5 mL sterile H_2_O and scraped using a 1 mL pipette tip. The plates were then allowed to rest for 10 min prior to a further 5 mL being applied. The liquid on the plates was then passed through a 10 mL syringe in which a small amount of sterile glass wool had been inserted into the barrel. The eluate from the syringe was collected in a 15 mL sterile plastic tube and centrifuged at 4000 g for 5 min. The pellet was resuspended in 5 mL sterile water and then diluted as required for subsequent counting using a haemocytometer.

The *Botryosphaeria* sp. pycnidia were counted by dividing the agar plates into eight and counting the pycnidia present on three representative sections from each plate. Three plates were used for each assay.

### *Sdh1* inactivation

The succinic semialdehyde dehydrogenase gene (*Sdh1*) disruption construct was created using the split-marker method [20]. p1 and p2 was used to amplify a 775 bp fragment 5′ of the *Sdh1* gene whilst p3 and p4 were used to amplify a 760 bp 3′ fragment. These 5′ and 3′ flanks were fused to the overlapping fragments of hygromycin cassette creating the constructs 5′sdh1KO-YG and 3′sdh1KO-HY. These constructs were then further amplified using standard proof-reading PCR conditions followed by PCR purification. 3 µg of each construct was then co-transformed into *S. nodorum* SN15 as previously described [19]. The resulting transformants were screened by PCR for homologous recombination using primers SdhKOscreenF and SdhKOScreenR, with a 2360 bp band representing the wild-type locus and a 3870 bp band showing a homologous recombination event. The s*dh1* complementation construct was created by amplifying the wild-type *Sdh1* gene as well as 1 kb upstream and 500 bp downstream of the open reading frame using the Sdh1compF and Sdh1compR primers. The resulting amplicon was fused to a phleomycin cassette as previously described [20] and transformed into the *sdh1-9* mutant. Gene copy number was determined by quantitative PCR as previously described [21]. The primer sequences are listed in Table S1.

In terms on nomenclature throughout the manuscript, *Sdh1* refers to the wild-type gene whilst Sdh1 denotes the protein. The inactive gene is denoted by *sdh1* (no capital ‘s’).

### Pathogenicity assays

The wheat line Grandin was grown for two weeks under natural day/night cycle at approximately 22°C in small pots containing vermiculite and 5 g of Osmocote slow release fertiliser. After two weeks, the plants were inoculated with spores at a concentration of 1×10^6^/mL containing 0.02% Tween 20 using an airbrush (Paasche, Chicago, USA). The plants were then covered for two days in the dark and scored visually as previously described at seven and 14 days post infection (dpi) [19].

### Statistical analysis

All statistical analyses were undertaken using the JMP7 package (SAS Institute). Statistical significance was determined using the Tukey–Kramer analysis.

## Results

### The identification and disruption of *Sdh1*

To identify the gene encoding a succinic semialdehyde dehydrogenase in *S. nodorum*, the Uga2 protein sequence from *S. cerevisiae* was used to interrogate the *S. nodorum* annotated genome sequence [22]. This approach identified the gene SNOG_00899 as being 54% identical to Uga2 with an E-value of 0. A reciprocal blast of the SNOG_00899 protein sequence against yeast identified Uga2 as the best match. A subsequent BlastP analysis of the SNOG_00899 protein sequence against the non-redundant database identified multiple genes putatively encoding fungal succinic semialdehyde dehydrogenases. Analysis of the protein sequence revealed multiple matches with known protein motifs associated with succinic semialdehyde dehydrogenase proteins (TIGR01780 and cd07103). On this basis, SNOG_00899 was renamed *Sdh1* and was chosen for subsequent analysis through targeted gene disruption.

The *Sdh1* gene disruption construct was amplified as described above and transformed into the wild-type *S. nodorum* strain SN15. Greater than 50 transformants were recovered, of which 24 were chosen for screening by growth on GABA as a sole nitrogen source. Four of the 24 transformants showed little or no growth when GABA was supplied as the sole nitrogen source. PCR screening of these four transformants resulted in an enlarged PCR amplicon across the *Sdh1* locus confirming the homologous integration of the disruption construct. These transformants were named *S. nodorum sdh1-9*, *12*, *21* and *24*. Quantitative PCR was used to confirm that each of the strains had only one copy of the hygromycin marker. Two of these homologous mutants, *sdh1-9* and *sdh1-21*, as well as an ectopic transformant, *S. nodorum Sdh1-2*, were selected for further characterisation (Figure S1). To confirm that the resulting phenotypes were due to the disruption of the *Sdh1* locus, a complementation construct was transformed back into the *sdh1-9* background restoring the ability of that strain to grow on GABA as a sole nitrogen source.

### Characterisation of *S. nodorum sdh1* strains

**Figure 2:**
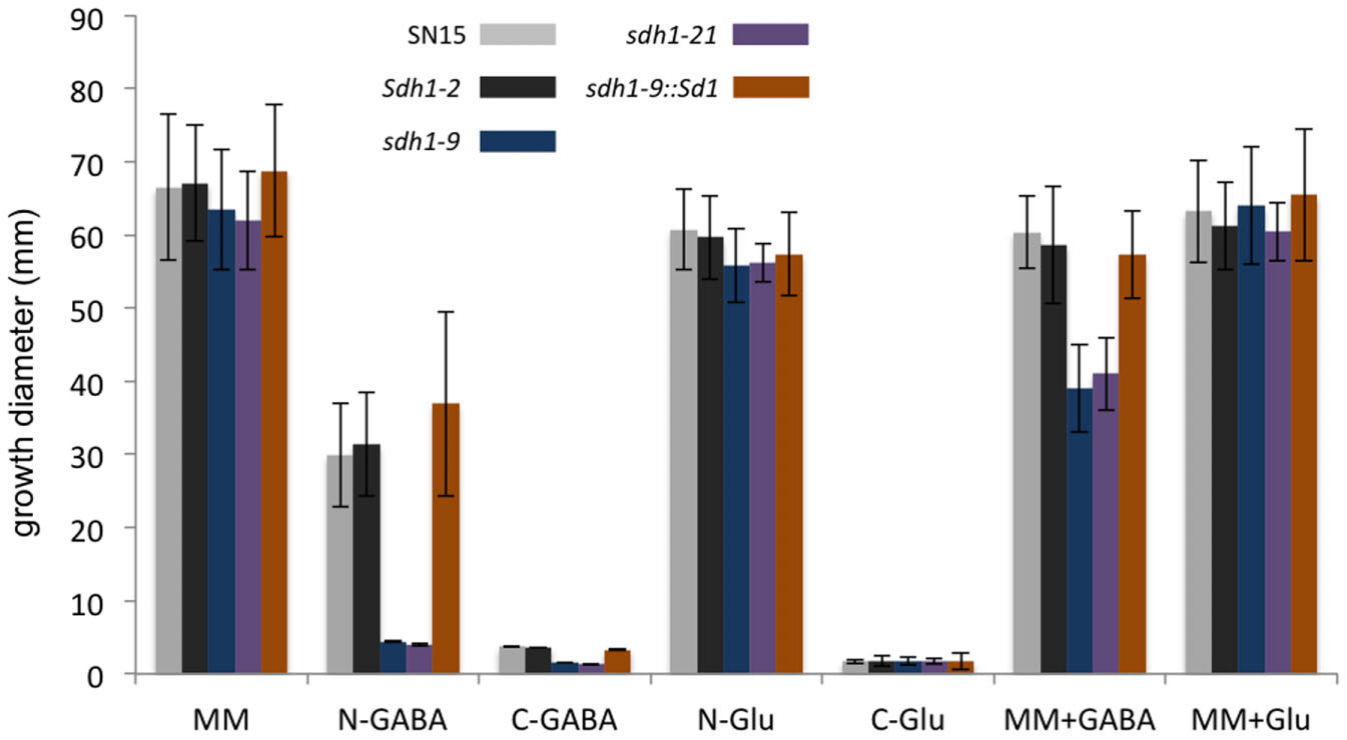
Plate growth assay. Colony diameter was measured after 18 days growth with n = 3. Standard error bars are shown. MM, minimal media; N-GABA, minimal media with GABA as the sole nitrogen source; C-GABA, minimal media with GABA as the sole carbon source; N-Glu, minimal media with glutamate as the sole nitrogen source; C-Glu, minimal media with glutamate as the sole carbon source; MM+GABA, minimal media with 1 mM GABA; MM+Glu, minimal media with 1 mM glutamate.

The role of Sdh1 was assessed during vegetative growth by measuring the growth of *sdh1-9* and *sdh1-21* strains on various defined media. The mutants displayed no difference in growth rates compared to *Sdh1* strains when grown in minimal media in the absence of GABA (Figure 2; Figure S2). When GABA was supplied as a nitrogen source rather than nitrate, the growth of the *sdh1* mutants was nearly 100-fold less compared to SN15 and the ectopic strain. None of the strains assayed grew strongly when GABA was supplied as a carbon source although the wild-type strain grew about 3-fold more than the *sdh1* strains. All of the strains grew comparably to each other when grown on glutamate as a carbon or nitrogen source. The growth of the strains harbouring an intact *Sdh1* gene was marginally affected by the inclusion of 1 mM GABA in complete minimal media. In contrast, the growth of the *sdh1* mutants was reduced by approximately 30% when GABA was included in the media. No difference in growth was observed when the mutants were grown on complex V8PDA media (data not shown).

**Figure 3:**
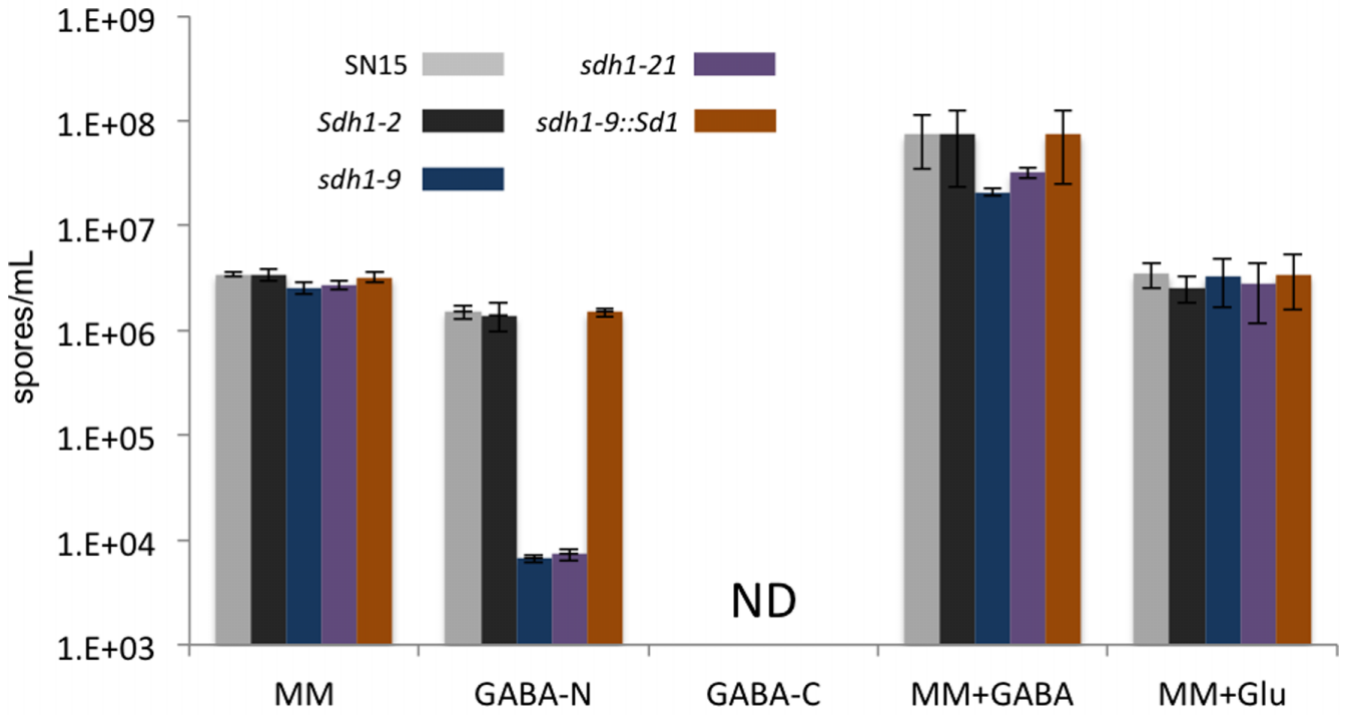
Sporulation assay for each of the strains grown under different nutritional conditions. MM, minimal media; N-GABA, minimal media with GABA as the sole nitrogen source; C-GABA, minimal media with GABA as the sole carbon source; MM+GABA, minimal media with 1 mM GABA; MM+GABA, minimal media with 1 mM GABA; MM+Glu, minimal media with 1 mM glutamate.

The strains were also assayed for their ability to sporulate in the media described above (Figure 3). There was no significant difference in the ability of strains to asexually sporulate on minimal media. The sporulation of the *sdh1* mutants on GABA as either a nitrogen or carbon source showed a similar trend to that observed for the growth assays as GABA proved to be a poor nitrogen source whilst no spores were detected in any of the strains assayed with GABA as a carbon source. When 1 mM GABA was added to minimal media as a supplement, the sporulation of all strains increased by five to 10-fold compared to the absence of GABA. This increase in total spores was particularly surprising for the *sdh1* strains given the negative impact on growth observed when GABA was included in the media as a supplement. Glutamate was also assayed as a supplement to determine whether or not the increased sporulation was due to more nitrogen being available. There was no increase in sporulation when glutamate was added to the media implying that the GABA sporulation effect was not due to nitrogen. Analyses of the resulting pycnidia from these sporulation assays revealed no phenotypic or viability differences when differentiated in the presence of GABA (data not shown).

**Figure 4:**
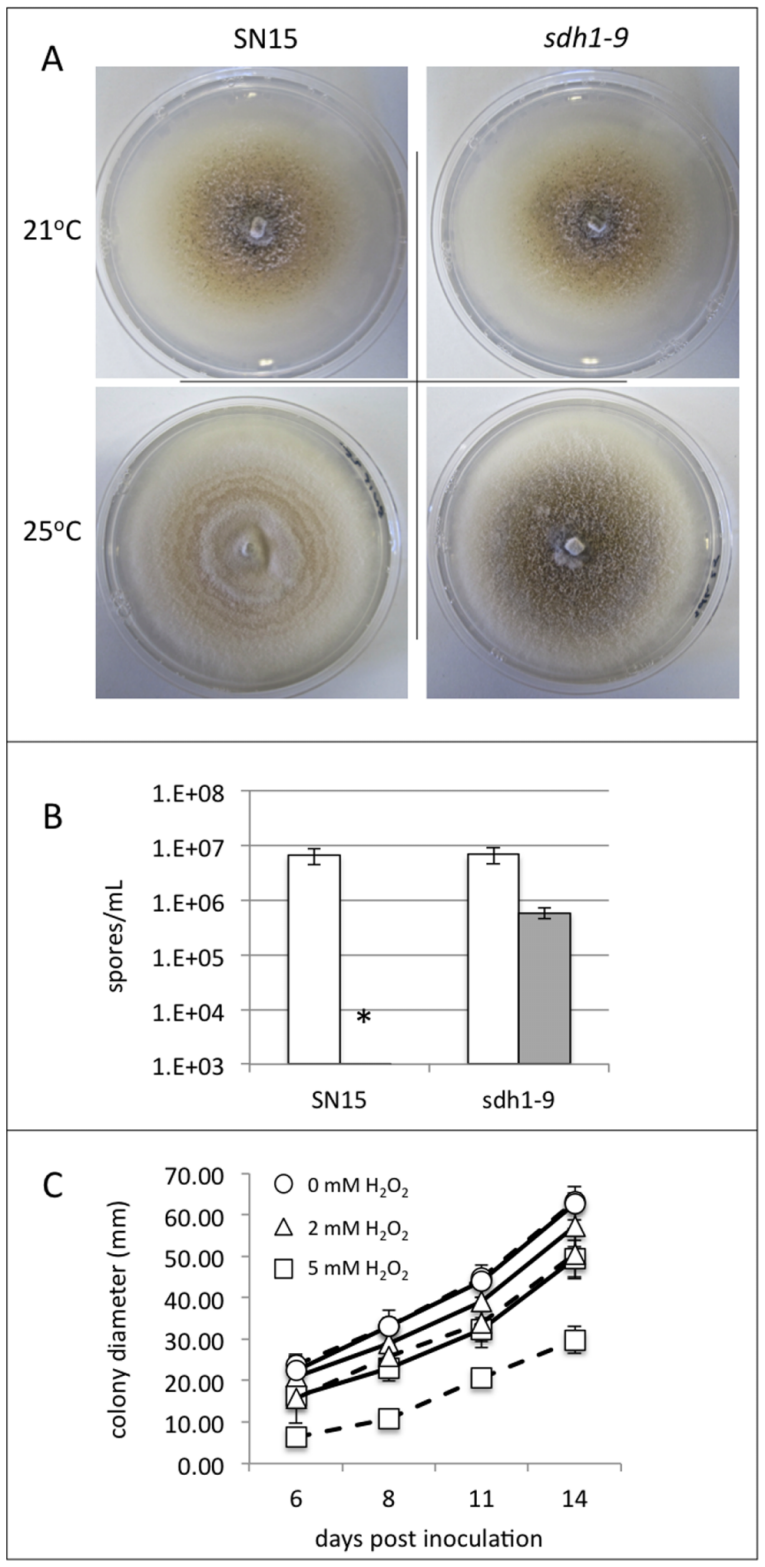
An analysis of the role of *Sdh1* during temperature and reactive oxygen species (ROS) stress. (A) Plate growth assays of the *S. nodorum* wild-type SN15 strain and the *sdh1-9* mutant at 21°C and 25°C. (B) Spores collected and counted from the plate sporulation in (A). (C) Growth assays of the SN15 (solid lines) and *sdh1-9* (broken lines) strains on different concentrations of H_2_O_2_.

The *sdh1* mutants were also tested for their resistance to a variety of stresses. The growth of the strains was examined during osmotic, pH, oxidative and temperature stresses. There was no difference on growth apparent in any of the strains when grown under different osmotic strengths or pH environments (data not shown). Similarly, the rate of growth was unchanged in the *sdh1* strains compared to SN15 at different incubation temperatures. There was however a strong phenotypic difference between the wild-type and mutant isolates when grown at 25°C, a high growth temperature for *S. nodorum* (Figure 4A). Incubation of the wild-type strain at the higher temperature resulted in a fluffy white appearance with no evidence of pycnidia. In contrast, the *sdh1* strains appeared much more similar to the phenotype observed at 21°C with pycnidia evident, although to a lesser degree at 25°C. Harvesting and counting of the spores on these plates revealed no significant difference in sporulation at 21°C when comparing the wild-type and *sdh1* strains (Figure 4B). At 25°C, the wild-type did not sporulate whilst a 10-fold decrease in sporulation was observed for the *sdh1* strains compared to growth at 21°C.

The susceptibility of the *sdh1* strains to reactive oxygen stress was assessed by including different concentrations of H_2_O_2_ in minimal media (Figure 4C). There was no significant difference in growth rate of the SN15 and *sdh1* strains up to concentrations of 1 mM H_2_O_2_. There was a small but significant decrease in growth of the *sdh1* strains compared to SN15 at 1 mM whilst the *sdh1* strains only grew to approximately 60% of SN15 at 5 mM H_2_O_2_ demonstrating that *Sdh1* has a role in detoxifying exogenous reactive oxygen species (ROS).

### *Sdh1* is required for complete pathogenicity

**Figure 5:**
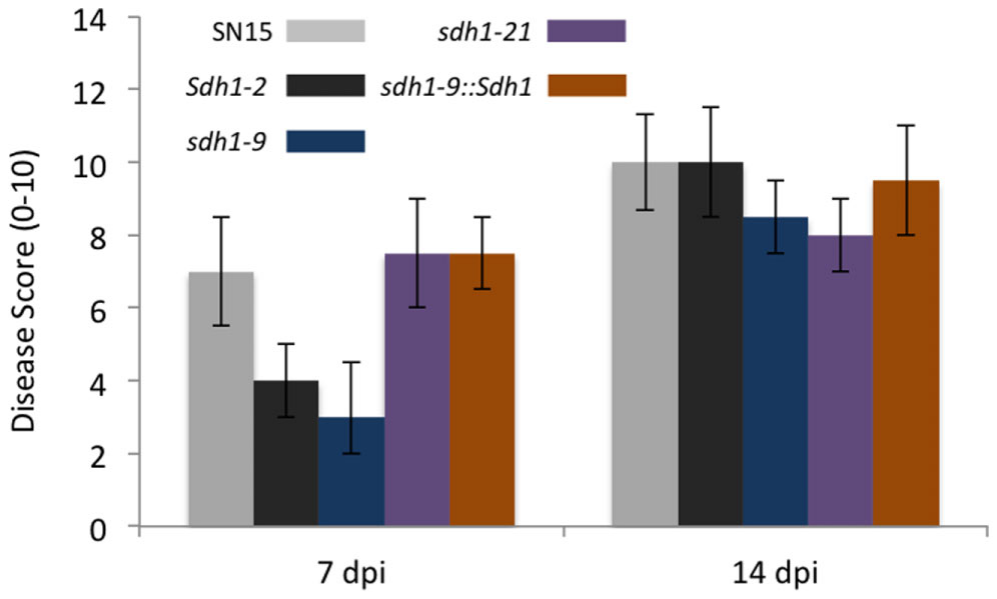
Pathogenicity scores for the wheat leaf infection assays. N = 5 and standard error bars are shown.

Spores generated from the *sdh1* mutants were used to inoculate wheat seedlings to determine the involvement of the gene in virulence. Disease scores recorded at seven dpi revealed the *sdh1* strains to be only half as pathogenic as the wild-type and ectopic isolates (Figure 5; Figure S3). A re-assessment of the disease after 14 dpi showed the disease scores of the *sdh1* mutants was closer to that of the wild-type, but still significantly less proving that S*dh1* does have a role in disease development.

### GABA promotes sporulation of *S. nodorum*

**Figure 6:**
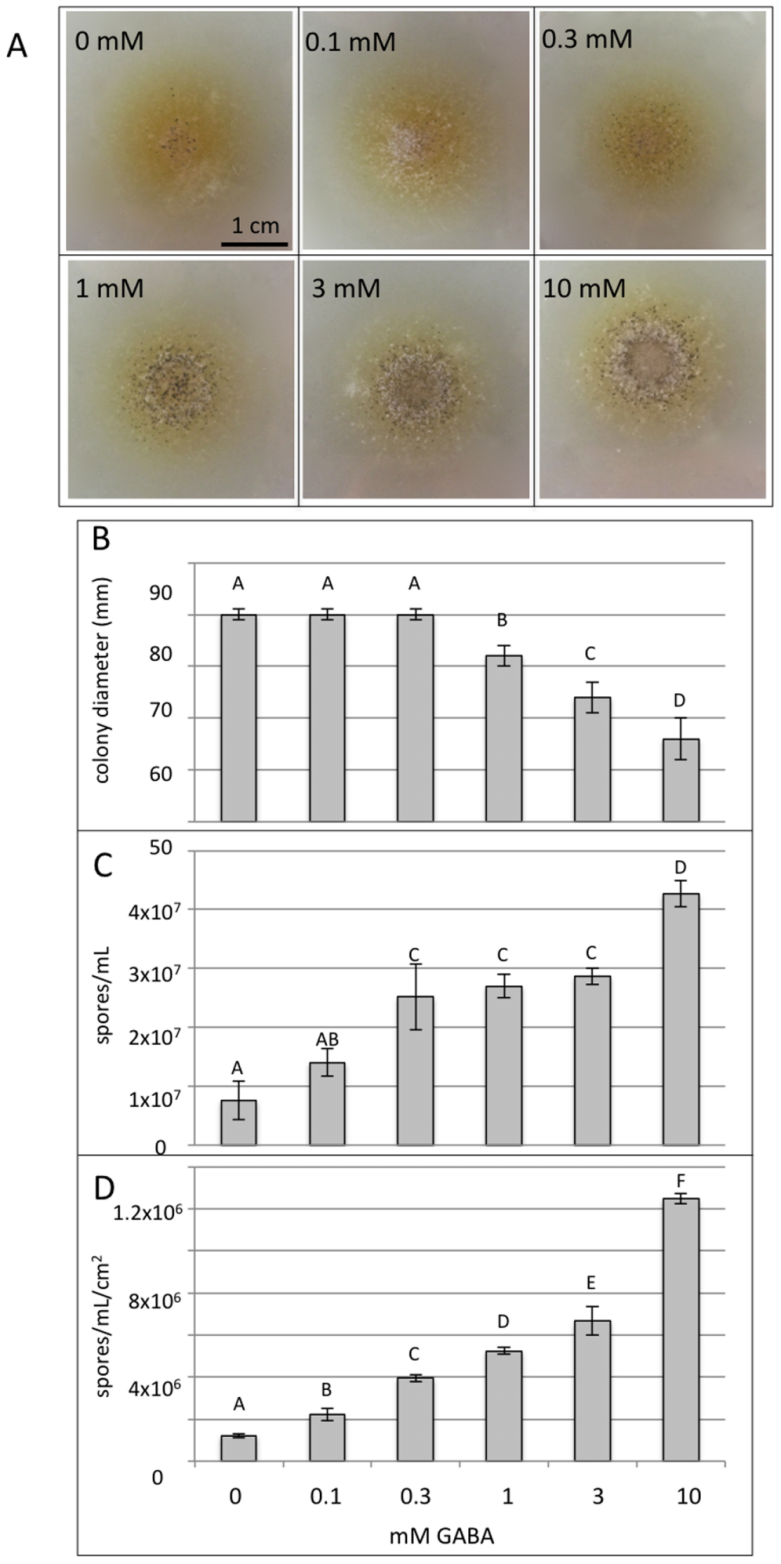
Growth and sporulation assays of *S. nodorum* SN15 growing in increasing GABA concentrations. (A) Images of *S. nodorum* growing at different GABA concentrations captured at eight days post-inoculation. Increasing levels of pycnidia (small dark spots) are clearly evident with higher concentrations of GABA. (B) Colony diameter; (C) Total number of spores per mL produced; (D) Number of spores produced divided by the area of colony growth. N = 6 and standard error bars are shown. The letters shown above each of the bars represent the statistical significance for that treatment with different letters representing treatments that are statistically different (p<0.05).

In the course of characterising of the *sdh1* strains, it was observed that the addition of GABA to minimal media (containing nitrate and sucrose) promoted sporulation. To investigate this further, *S. nodorum* SN15 was plated out onto varying concentrations of GABA to determine if the positive effect on sporulation was dose dependent. An equal number of spores were plated onto the centre of a standard minimal media plate containing 0, 0.1, 0.3, 1, 3 or 10 mM GABA (Figure 6). The plates were then allowed to grow for 18 days prior to the spores being harvested and counted.

The first observation was that the increasing concentrations of GABA appeared to inhibit growth. There was no change in total growth observed up to 0.3 mM GABA, however higher concentrations restricted the rate of growth. Spore counts from the same plates revealed a positive correlation of GABA concentration and sporulation. The addition of 0.3 mM GABA was required to see a significant increase in sporulation with 10 mM resulted in an eight-fold increase in the number of spores produced per plate. When the reduced growth area of the cultures growing on higher levels of GABA was considered, the supplementation of the media with 10 mM GABA resulted in a 10-fold increase in sporulation.

**Figure 7:**
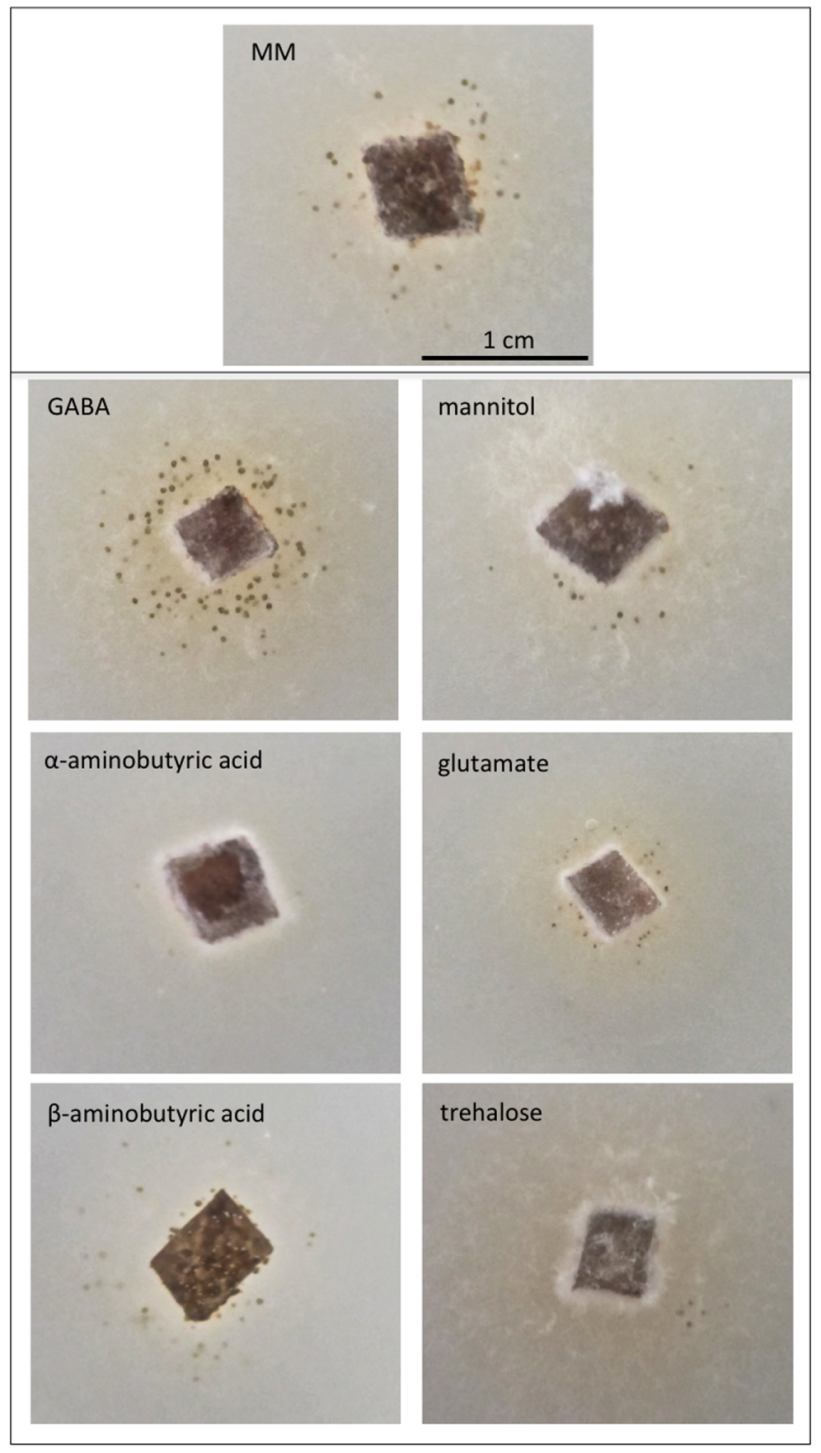
Plate growth assays of *S. nodorum* SN15 growing on minimal media supplemented with different compounds. All compounds were added to a final concentration of 1

Minimal media was supplemented with isomers of GABA and other compounds known to induce sporulation in *S. nodorum* to see if this positive effect on sporulation was specific to GABA (Figure 7). With the exception of GABA, none of the compounds assayed resulted in any increase in sporulation. A decrease in sporulation was observed with the addition of α-aminobutyric acid to minimal media, probably reflecting the poor growth of *S. nodorum* in the presence of this GABA isomer.

### The GABA effect on sporulation is not specific to *S. nodorum*

**Figure 8:**
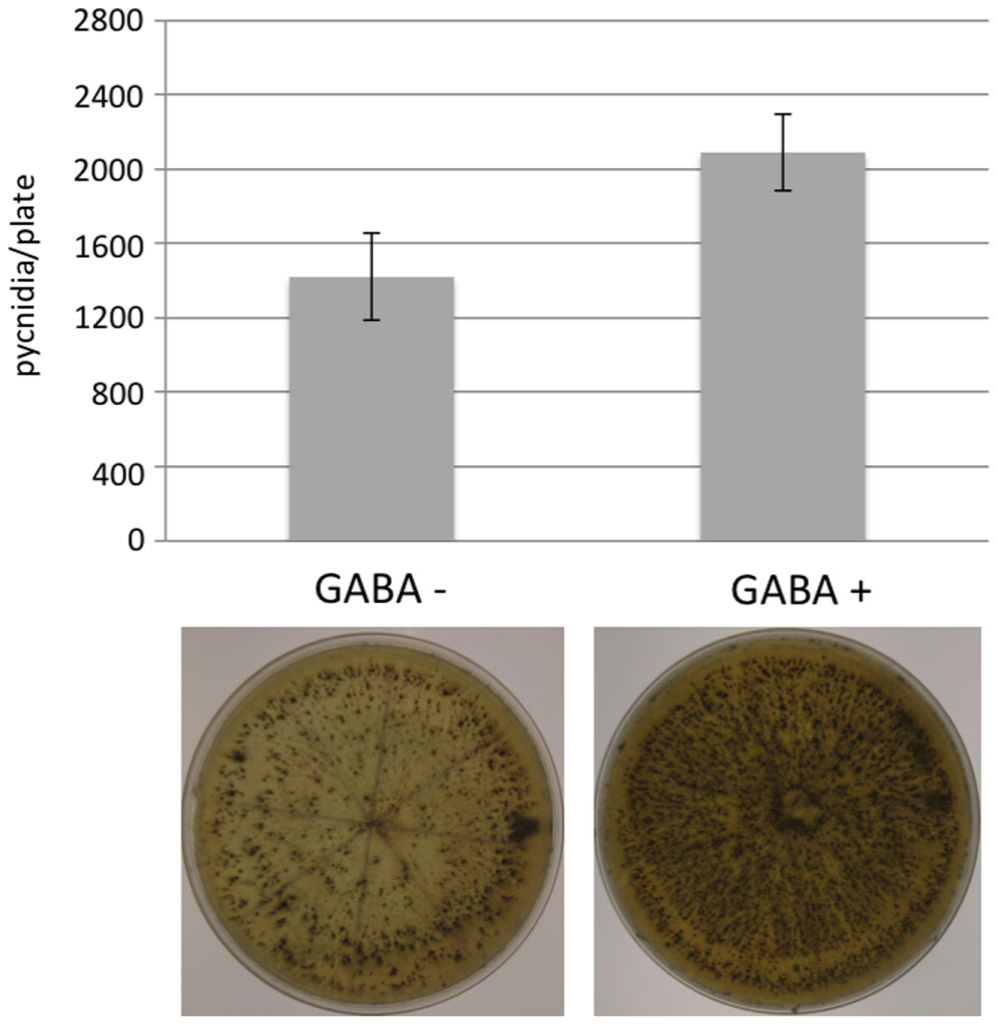
*Botryosphaeria* sp. sporulation assays in the presence and absence of 1 mM GABA.

To determine if the positive effect of GABA on sporulation was specific to *S. nodorum*, we examined its effect on the ability of a related fungus *Botryosphaeria* sp. to asexually differentiate. In the absence of GABA, an average of 1400 pycnidia were counted per plate shortly after exposure to near-UV light (Figure 8). The addition of 1 mM GABA to the same growth media resulted in an approximate 47% increase in the number of pycnidia counted (average 2064 per plate). The data suggest that the effect of GABA on sporulation is not specific to *S. nodorum*.

## Discussion

Since the discovery of GABA metabolism in fungi, its role has been the subject of several hypotheses, but with little supporting evidence. Amongst these roles, it has been speculated that GABA metabolism in phytopathogenic fungi is important for disease. This idea was proposed by Solomon and Oliver [23] when they reported millimolar levels of GABA present in the tomato apoplast during a compatible interaction with the biotrophic fungus *Cladosporium fulvum*. In a subsequent study, the same authors demonstrated that the pathogen genes required to metabolise GABA were expressed during infection [24]. This lead to the suggestion that perhaps *C. fulvum* was able to manipulate the host in order to provide GABA as a nitrogen source during infection, although there has been no data published since to support this [16]. Consequently, to better understand the role of GABA in filamentous fungi, and in particular phytopathogens, we analysed its metabolism in the wheat pathogen *S. nodorum*.

The first observation from this study was that *Sdh1*, the gene encoding succinic semialdehyde dehydrogenase in *S. nodorum*, plays a role in stress response and pathogenicity. Under standard growth conditions, the growth rate and phenotype of *S. nodorum* mutants lacking *Sdh1* appeared unaffected compared to SN15. When GABA was supplied as a nitrogen source, the *sdh1* strains grew poorly as expected, as only a small amount of nitrogen would have been available for growth through alanine as a result of GABA transaminase. Comparable trends were observed when sporulation was assayed with the *sdh1* mutant producing fewer spores than the wild-type when GABA was supplied as either a nitrogen or carbon source. These data fit the current dogma in that GABA can only be metabolised via a transamination and oxidation back to succinic acid.

It was apparent though from the growth assays that *Sdh1* has a role in enabling *S. nodorum* to successfully respond to certain stresses. Upon exposure to 5 mM H_2_O_2_ the *sdh1* mutants grew poorly compared to SN15 implying that the metabolism of GABA is involved in protection against ROS. Previous studies in *S. cerevisiae* and *M. oryzae* have demonstrated a role for GABA metabolism in resisting oxidative stress. In yeast, strains lacking either UGA5 (succinic semialdehyde dehydrogenase) or UGA1 (GABA transaminase) show increased susceptibility to higher concentrations of H_2_O_2_ directly implying that the catabolism of GABA has a role in ROS detoxification [25]. The authors speculated that the basis of this resistance is through the production of NADPH by UGA5 during the oxidation of succinic semialdehyde. In *M. oryzae*, MoSSADH was found to be regulated by a homologue of the YAP1 transcription factor, which is critical in regulating the response to ROS. Subsequent deletion of the MoSSADH gene revealed that the resulting mutants were susceptible to reactive oxygen stress. Whilst it is possible that decreased NADPH levels may be responsible for the susceptibility to ROS in MoSSADH mutants (although NADPH was not measured in the mutants), the mutants also displayed attenuated secreted peroxidase activity. One could simply speculate that reduced peroxidase activities were more likely to be responsible for the increased ROS susceptibility than reduced NADPH levels. It was unclear though how MoSSADH regulates peroxidase levels.

Another interesting phenotype displayed by the *S. nodorum sdh1* mutants was their ability to better cope with higher than normal growth temperatures. At 21°C, the phenotypes of the *sdh1* and SN15 strains appeared identical. At 25°C, the growth rate of the SN15 strain was comparable to that at 20°C, but appeared whiter in colour and more filamentous with no evidence of pycnidia or sporulation. In contrast the *sdh1* strains displayed a more comparable phenotype to the growth observed at 20°C. Pycnidia were also evident, and although less than at 20°C, viable spores could be extracted. This is the first evidence that the GABA shunt plays a role in temperature stress in fungi, although the mechanism underlying this is unclear.

Perhaps not surprisingly, after demonstrating the increased susceptibility of the *sdh1* strains to different stresses, the mutants were less pathogenic than wild-type isolates. At seven dpi, the *sdh1* strains were about half as virulent as the wild-type, increasing to about 70% virulence at 14 dpi. It is highly likely that the pathogen would be subjected to a variety of stresses during infection. It has been previously established that elevated ROS levels are a result of the photosynthetic collapse caused by the effector SnToxA [26]. The increased susceptibility of the *sdh1* mutants to H_2_O_2_ would suggest that the mutants may struggle to deal with the high *in planta* H_2_O_2_ levels resulting in slower growth. In contrast, deletion of MoSSADH in *M. oryzae* resulted in a completely non-pathogenic strain [18]. The *M. oryzae* mutants were unable to penetrate the cuticle effectively nor grow vegetatively within the leaf. *Stagonospora nodorum* and *M. oryzae* are contrasting pathogens with different penetration and infection mechanisms. There are multiple examples of genes that are involved in the pathogenicity of *M. oryzae* that do not share a similar role in *S. nodorum* [7], [27], [28]. The data presented in this study would suggest that *Sdh1* maybe another.

Of particular interest was serendipitous observation of the effect of GABA on the growth and sporulation of *S. nodorum* when added to complete minimal medium (i.e. containing sucrose and nitrate). Increasing concentrations of GABA in the minimal medium clearly inhibited the growth of *S. nodorum*, both in wild-type and *sdh1* strains. This impairment of growth is likely to be due to the increased levels of succinic semialdehyde which itself could be toxic [15].

Perhaps the more striking observation of GABA supplementation was that *S. nodorum* produced nearly 10-fold more spores on MM when supplemented with GABA. This was surprising when considering that sporulation was unaffected when GABA was supplied as a sole nitrogen source; the GABA effect was only evident when the compound was added to complete MM. GABA has been previously described to promote conidiation in *Penicillium marneffei*, although no further details were reported [29]. The positive effect of GABA on asexual sporulation was dose dependent, with increased GABA levels leading to higher spore production. Isomers of GABA and other compounds previously associated with sporulation in *S. nodorum* were also assayed with no effect observed other than a growth defect on β-aminobutyric acid (BABA). Previous reverse genetic studies have demonstrated the requirement for the mannitol and trehalose metabolic pathways for asexual sporulation [5], [7]. However, these compounds do not increase sporulation when included in complete minimal media suggesting the mechanisms behind GABA-induced sporulation are independent of mannitol and trehalose.

The obvious question from this data is why does the presence of GABA increase the rate of sporulation? Perhaps some light can be shed from the data on the *sdh1* mutants. The inclusion of 1 mM GABA in minimal media significantly affected the growth of the *sdh1* mutants, which as discussed above, was likely due to the increased accumulation of succinic semialdehyde. However, as for the wild-type strain, the presence of GABA also stimulated spore production in the *sdh1* mutants. There are two possible reasons for this. Firstly, GABA could be metabolised via a route other than through succinic semialdehyde, and that this alternative pathway may contribute to sporulation. However the inability of the *sdh1* strains to be able to grow on nitrogen as a sole nitrogen source would suggest this is unlikely.

Another possibility is that that whilst GABA is metabolised through the conventional pathway (and thus leading to the growth defect in the *sdh1* strains), it may also have another role in inducing sporulation. Precisely how GABA would fulfil this alternative role is unclear but there is existing evidence that it can act as a signalling molecule. Chevrot and colleagues discovered that GABA stimulated the inactivation of the N-(3-oxootanoyl)homoserine lactone quorum-sensing signal secreted by *Agrobacterium tumefaciens* [30]. Quorum sensing is a mechanism of cell-to-cell communication predominantly undertaken by bacteria [31], [32]. Multiple studies have shown that quorum sensing is involved in the successful association of bacteria with eukaryotic organisms [33], [34]. The concept of small diffusible signalling molecules triggering sporulation in filamentous fungi is not without precedent. Studies by Adams and colleagues identified that FluG in *Aspergillus nidulans* is involved in the secretion of a small molecule that accumulates externally to induce sporulation [35]. Whilst it has been shown since that the molecule in *A. nidulans* is not GABA [36], our data does show that exogenous GABA does stimulate sporulation. Studies are underway to dissect this intriguing phenomenon further.

## Supporting Information

Figure S1 (A) PCR amplification of *Sdh1* locus using the SdhKOscreenF/R primers (Supplementary Table S1). A band of 2321 bp represents the wild-type locus and 4243 bp the *sdh1* mutants. (B) Screening of the different strains for the presence of hygromycin (528 bp) and phleomycin (1998 bp). For both (A) and (B), lane 1 – 1 kb ladder, lane 2 – *S. nodorum* SN15, lane 3 – *sdh1-9*, lane 4 – *sdh1-21*, lane 5 – *Sdh1-2* and lane 6 - *sdh1-9::Sdh1*. (C) A histogram representing the number of copies of the hygromycin and phleomycin relative to that of γ-actin. The primers for these listed in Supplementary Table S1. The *S. nodorum* strain *mpdmdh102* was included as a positive control as it has been previously demonstrated to have one copy each of hygromycin and phleomycin (Solomon PS, Ipcho SVS, Hane JK, Tan K-C, Oliver RP (2008) A quantitative PCR approach to determine gene copy number. Fungal Genetics Reports 55: 5–8.).
(TIFF)

Figure S2 An example of the growth differences of the *sdh1* strains growing on either nitrate (A) or GABA (B) as a nitrogen source.
(TIFF)

Figure S3 Representative pots infected with *S. nodorum* SN15 (left) or the *sdh1-9* strain (right). Images were captured at 7 dpi. The red arrows show examples of heavily infected leaves.
(TIFF)

Table S1 Primer sequences.
(DOCX)
