# Supplementary figures and images for: Characterising the Role of GABA and Its Metabolism in the Wheat Pathogen Stagonospora nodorum
Source: PLoS One. 2013 Nov 12;8(11):e78368. doi: 10.1371/journal.pone.0078368 (PMC3827059; doi:10.1371/journal.pone.0078368)

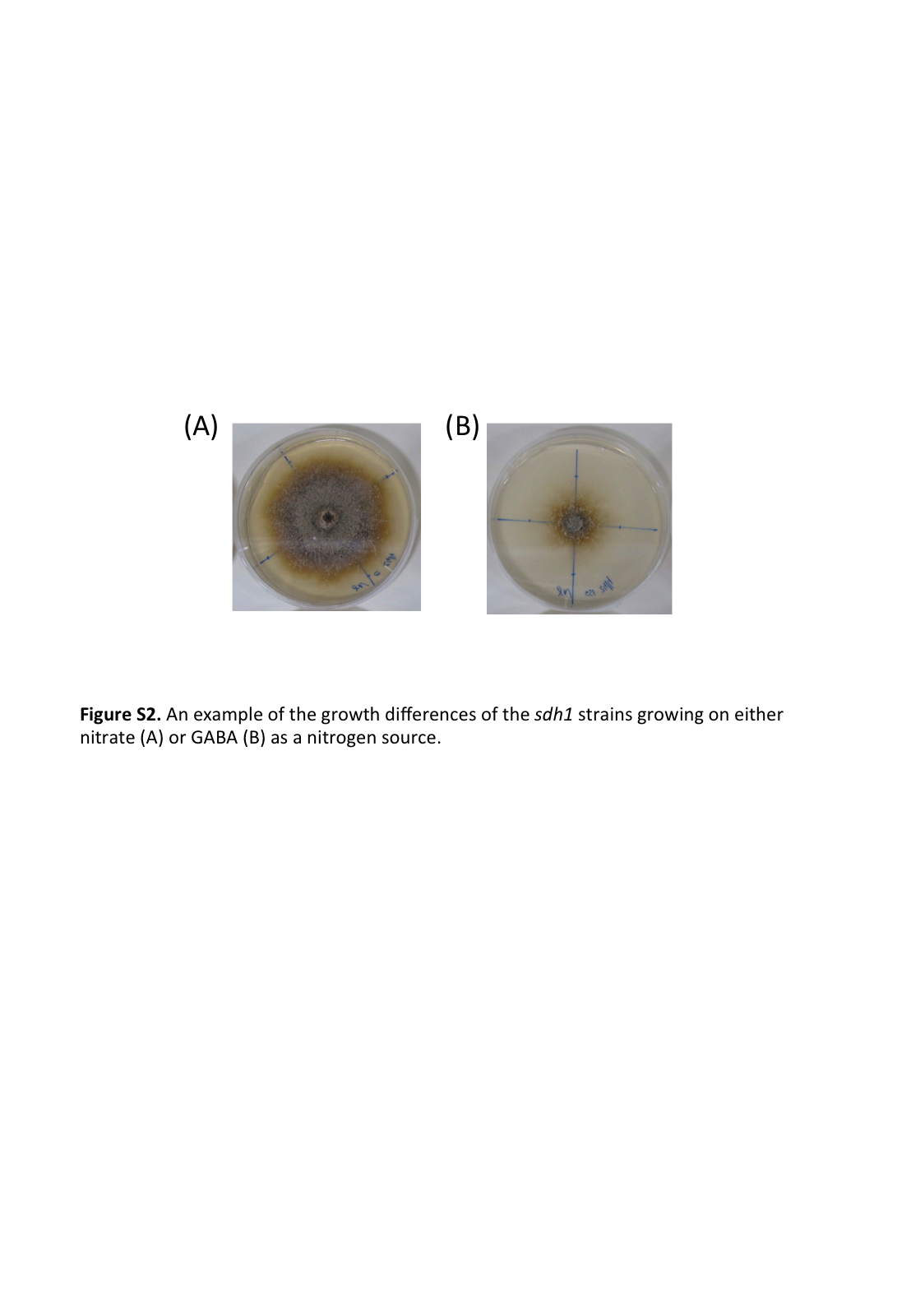

Supplement: Figure S2 — An example of the growth differences of the sdh1 strains growing on either nitrate (A) or GABA (B) as a nitrogen source. (TIFF) [file pone.0078368.s002.tif]

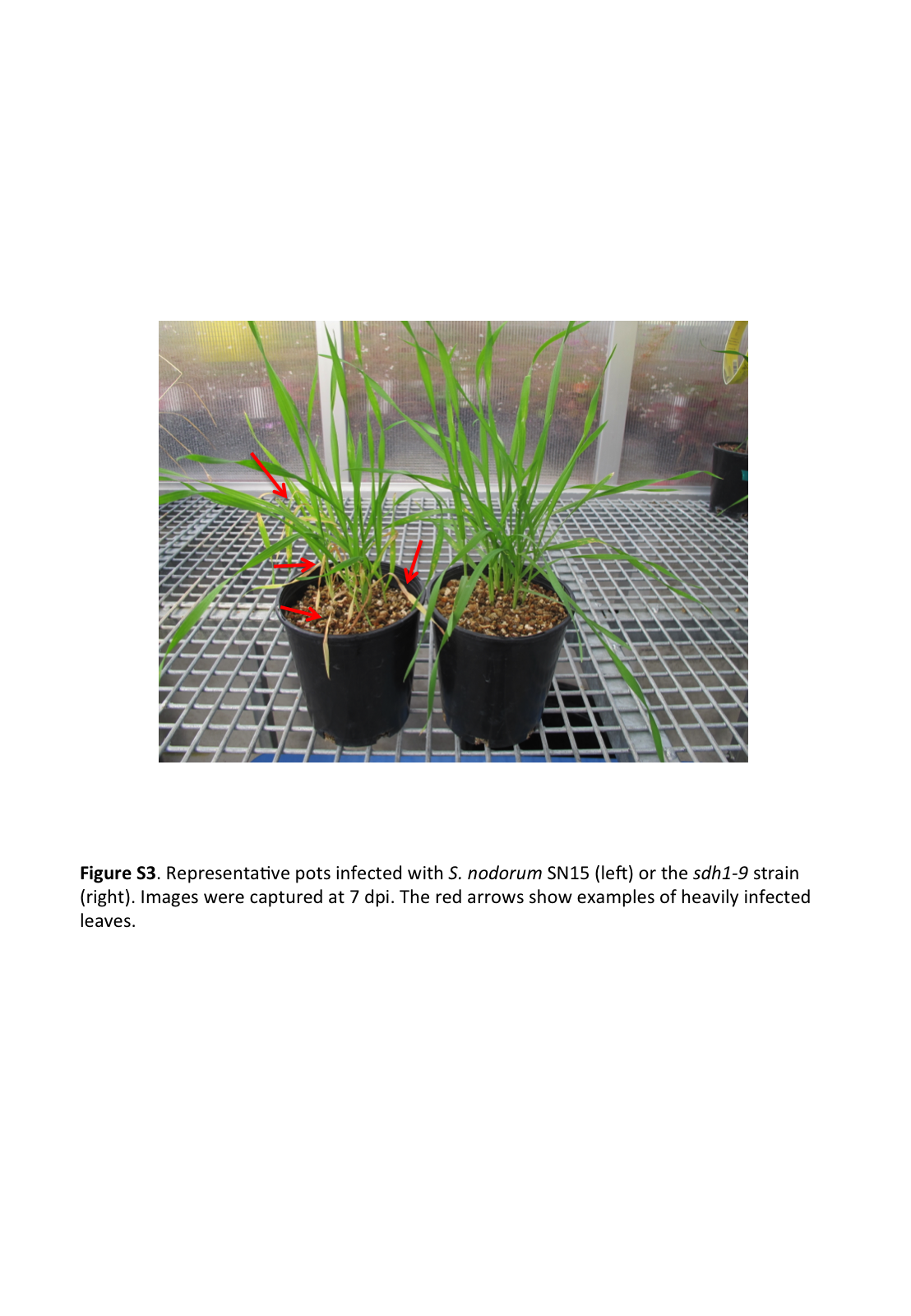

Supplement: Figure S3 — Representative pots infected with S. nodorum SN15 (left) or the sdh1-9 strain (right). Images were captured at 7 dpi. The red arrows show examples of heavily infected leaves. (TIFF) [file pone.0078368.s003.tif]
