# Supplementary material for: Characterising the Role of GABA and Its Metabolism in the Wheat Pathogen Stagonospora nodorum
Source: PLoS One. 2013 Nov 12;8(11):e78368. doi: 10.1371/journal.pone.0078368 (PMC3827059; doi:10.1371/journal.pone.0078368)
Supplement: Table S1 — Primer sequences. (DOCX) [file pone.0078368.s004.docx]

**Table S1.** Primer sequences.

| **Primer name** | **Sequence** |
| --- | --- |
| p1 | ATGTAACCGATGCCGACTT |
| p2 | GTACTGTGTAAGCGCCCACTCTCGCATGGTGGATGTAGAG |
| p3 | TTGGGAGCTCGGTATAAGCGCCTTATACTGCGGCTCCTG |
| p4 | GATAGCGAGCGGCTTAATAC |
| SdhKOscreenF | TAGGCGGCCATGACAATCAG |
| SdhKOScreenR | ACCCGGACAACTTCAGGAAC |
| Sdh1compF | ATCCAGCTTGCAAAGACG |
| Sdh1compR | GCTCCTCGCCCTTGCTCACCATAGGGATCTTGAGCAGGTGA |
